# Influence of Modified Carbon Black on Nylon 6 Nonwoven Fabric and Performance as Adsorbent Material

**DOI:** 10.3390/nano12234247

**Published:** 2022-11-29

**Authors:** Marlene Andrade-Guel, Pamela Yajaira Reyes-Rodríguez, Christian J. Cabello-Alvarado, Gregorio Cadenas-Pliego, Carlos Alberto Ávila-Orta

**Affiliations:** 1 Centro de Investigación en Química Aplicada, Saltillo 25294, Mexico; 2 Instituto de Ciencias Humanidades Lic. Salvador González Lobo, Universidad Autónoma de Coahuila, Saltillo 25125, Mexico; 3 CONACYT—Centro de Investigación en Química Aplicada, Saltillo 25294, Mexico

**Keywords:** modified carbon black, nylon 6 nonwoven fabric, uremic toxins, chronic renal kidney

## Abstract

The number of chronic kidney disease (CKD) persons continues to rise in Mexico. They require renal replacement therapy, and in the absence of it, hemodialysis is the major option for their survival. The uremic toxins present in the blood are removed by hemodialysis, which involve membranes. In this study, nonwoven fabrics with modified carbon black nanoparticles in a matrix polymer of Nylon 6 were obtained and evaluated as an adsorbent material of uremic toxins. All nonwoven fabrics were characterized by FTIR, XRD, TGA, SEM, and contact angle measurements and were evaluated as an adsorbent material for the urea toxin and as an albumin retainer. The findings suggest their potential application as a hemodialysis membrane. Nanocomposites had a higher hydrophilic characteristic compared to pure Nylon 6. The average diameter size of the fibers was in the range of 5 to 50 μm. All nanocomposites nonwoven fabrics showed high removal percentages of inulin in a range of 80–85% at 15 min of contact. Most Ny6 Zytel/CB nanocomposites showed a high percentage of urea removal (80 to 90%).

## 1. Introduction

Chronic kidney disease (CKD) is one of the leading causes of mortality worldwide, with the incidence rate of this disease increasing significantly in recent years. This condition results from the progressive decrease in renal filtration and excretion capacity, causing an increase in the concentration of uremic toxins in the bloodstream producing unwanted side-effects [1,2]. Today, hemodialysis is an essential treatment for the elimination of toxic compounds present in patients with chronic kidney disease. The main component of the hemodialysis equipment is a semi-permeable membrane that allows the elimination of low-molecular-weight solutes such as urea, creatinine, and uric acid through diffusive and convective transport [3,4].

The mechanism of removal of uremic toxins is well known: it is carried out through an adsorption process that can be chemical or physical. Chemical adsorption is a process resulting from a chemical bond between adsorbate molecules and specific surface locations on a material, whereas in the physical adsorption, the interaction forces are van der Waals. During the adsorption process, the molecules are held loosely on the adsorbent surface and can be easily removed. This process is different to the absorption process, where the molecules are entirely dissolved or diffused in the absorbent to form a solution. Once dissolved, the molecules cannot be separated easily from the absorbent. The adsorption isotherms mechanism and the interactions between the adsorbent and the adsorbate are explained by different mathematical models [5]

The removal of medium and high molecular weight is a limited process for conventional processes. There are several different types of adsorbents reported in the literature, including activated carbon, resins, carbon nanotubes, graphene, mesoporous silica, zeolites, and metal–organic frameworks. Nanoporous adsorbents with high surface area have offered great potential as biomaterials for CKD. Activated carbon and zeolite have been the most commonly used adsorbents as they can adsorb a variety of uremic toxins and are relatively inexpensive compared to other types of materials. Both are common adsorbents applied in purification to increase ultrafiltration properties. AC has a high adsorption capacity of uremic toxins but is not selective in size and can simultaneously remove other useful molecules. The deficiency in the different materials used in hemodialysis stimulates the development of new adsorbent materials, with high adsorption capacity, good selectivity, biocompatibility, and low cost [6,7]. 

The most promising adsorbent materials are those carbon-based materials (carbon nanomaterials), due to their high performance and relatively low cost. These materials have demonstrated a high capability of adsorption for different organic and inorganic compounds and heavy metal ions [8,9,10]. An excellent review article was recently published with specific information on adsorbents used in the removal of uremic toxins, traditional adsorbents, and certain special adsorbent systems such as molecularly imprinted polymer and mixed matrix membranes [11]

Carbon black (CB) is a fine black powder of nearly pure elemental carbon. It has many applications: its single largest use is as a reinforcing agent in vehicle tires and also in inks, paints, plastics, and coatings. The fundamental properties of carbon black determine the performance of the application. These include: particle size, structure, porosity, surface chemistry or surface activity, and physical shape. Porosity is an important property in adsorption applications. Carbon black tends to have a high degree of porosity, which makes it extremely attractive [12]. There are many reports about the adsorption capacity of CB to remove water vapor, NH_3_, and CO_2_ from the atmosphere [13,14], as well as for protein and amino acid separation [15,16]. CB has been used as an adsorbent material in the removal of uremic toxins, mainly for its high adsorption–desorption capacity. Molecular adsorption of CB depends largely on its surface area, high microporosity, and material source [17,18].

Nylon 6 does not dissolve in water or conventional organic solvents, does dissolve in phenol, cresol, and formic acid, and melts at 236 °C. Its resistance to stress is much higher than those of wool, silk, rayon, or cotton. Nylon 6 is used to produce filaments much finer than conventional fibers. Its potential applications allow its use in parachutes, insect nets, and surgical sutures and is mainly used in the textile industry.

Nylon fibers alone do not have antimicrobial properties; therefore, to acquire those properties, the fibers have to be impregnated or modified with antibacterial substances. There are reports in the literature of the development of textiles (nylon fibers) with bactericidal, antiviral, and fungicidal activity [19,20,21]. There are also reports of nanocomposites of Nylon 6 with antimicrobial properties, involving other polymer matrices and nanoparticles as reported by Mahdavi et al. [22]. Among the methodologies commonly used for the manufacture of textile fibers are electrospinning, forced spinning, nanospider technology, biocomponent spinning, and melt-blowing [23,24,25,26,27,28]. In this work, specifically, the nonwoven fabric of Nylon 6 and its respective composites were manufactured by melt-blowing, which is the process that uses an airstream directed across the path of the falling filament just below the spinneret exits to break the filaments into relatively short lengths, and the resultant fibers are collected on a moving belt, pressed, and sintered into a sheet. The literature reports several investigations aimed at the production of membranes through the technique of molten blowing for different applications such as for oil/water separation, filtering respiratory protective devices, and solvent-vapor-sensitive nonwoven fabric [26,29,30]. Cellulose and their derivatives were used for manufacturing the first membranes of the dialyzer, but these materials presented low permeability. The second generation of hemodialysis membranes were manufactured with synthetic polymers such as polysulfone (PS), polyurethane sulphone (PES), and polyacrylonitrile (PAN) [31]. In the literature, manufactured hemodialysis membranes composed of different polymeric matrices have been reported [32,33,34], as well as some other manufactured polymer hollow fibers [35,36]

In this research, the manufacturing of nonwoven fabrics of Nylon 6 with modified carbon black nanoparticles is presented. Modification of CB was carried out with amino acids compounds such as 4-aminobutiric acid. The incorporation of modified CB and its influence on its structural, thermal, and morphological properties of Nylon 6 were investigated. In addition, the performance in the adsorption of uremic toxins such as urea, albumin, and inulin was assessed.

## 2. Materials and Methods

### 2.1. Materials

Nylon 6 (DuPont Zytel^®^ 7301 NC010) was used as polymer matrix. Carbon black (VULCAN XC-72-CB, diameter 15 nm, purity 99%) and 4-aminobutanoic acid (≥99% purity) were supplied by Sigma Aldrich (Saint Louis, MO, USA).

### 2.2. Chemical Modification of Carbon Black (CB) with Amino Groups

Carbon black modification with 4-aminobutanoic acid was made following the methodology already reported in previous studies [37]. The MCBN contained a 30.5% modification with respect to unmodified carbon back.

### 2.3. Nanocomposite Preparation

The nanocomposites were prepared using the methodology of Cabello-Alvarado et al. [38,39]. Nylon 6/modified carbon black nanoparticles (MCBNs) nanocomposites were obtained with several percentages by weight of MCBNs. The weight percentages used were 0, 0.25, 0.5, 0.75, 1.0, 2.0, and 4.0 of MCBNs. Table 1 shows the identifications of each of the nanocomposites obtained. It is important to note that, for the first time, the preparation of Nylon 6/MCBN nanocomposite nonwoven fabric with high weight percentages of MCBNs (4.0 wt.%) was reported.

### 2.4. Nonwoven Fabric Preparation

Nylon 6/MCBN nanocomposite nonwoven fabric was made by fiber extrusion technology (FET-UK) pilot machine equipment. The processing parameters were: 230 °C extrusion zone 1, 240 °C extrusion zone 2, 245 °C extrusion zone 3, 245 °C extrusion zone 4, 245 °C flange zone, 245 °C melt pump zone, 245 °C dual heat zone, 245 °C melt blow adapter zone, and 215 °C melt blow hot air zone, which were used for obtaining the nonwoven fabric [40,41].

### 2.5. Characterization Techniques

#### 2.5.1. Thermogravimetric Analysis (TGA)

A thermogravimetric analysis (TGA) Q500 model (TA instruments., New Castle, PA, USA) was utilized for analyzing Ny6 and all composites of Ny6 Zytel/CB. The operating conditions consisted of a heating rate of 10 °C/min and an air atmosphere with a gas flow of 50 mL/min. The samples run were performed from 300 °C to 600 °C in an N_2_ atmosphere. Once 600 °C was reached, the N_2_ atmosphere was switched to O_2_ atmosphere for a better combustion of organic components.

#### 2.5.2. Differential Scanning Calorimetry (DSC)

This analysis was performed on a DuPont Instruments 951 analyzer, at a heating rate of 10 °C/min at a temperature range from 40 °C to 300 °C. Through a primer heating cycle, the thermal history of the samples was eliminated before obtaining the second run.

#### 2.5.3. X-ray Diffraction (XRD)

For this test, a Rigaku Smartlab diffractometer with a scanning interval in the 2θ scale from 5 to 80° and a scan speed of 0.02% was used. The radiation employed was copper Kα with a wavelength of 1.54 Å, and values of 40 mA and 40 kV were used for intensity and voltage, respectively, with a stability of 0.01%8h.

#### 2.5.4. Fourier Transform Infrared Spectroscopy (FTIR)

To obtain ATR spectra of samples of Nylon 6 and its synthetized nanocomposites, a Magna Nicolet 550 spectrometer was used.

#### 2.5.5. Scanning Electron Microscopy (SEM)

The morphology and energy dispersion spectroscopy (EDS) were examined by means of the scanning electron microscopy model JEOL JSM-7401F. The surface of the analyzed samples was covered with a layer of approximately 10 nm of the conducting material gold-palladium.

### 2.6. Contact Angle (Wettability)

For this study, a Ramé-Hart goniometer model 100–00 was used. Nonwoven fabric of pure Nylon 6 and nonwoven fabric of Nylon 6 with MCBNs at different concentrations were evaluated for determining their wettability to water (1 μmL). All analyses were carried out at 25 °C and the samples of nonwoven fabric had a size of 1 × 8 cm.

### 2.7. Toxin Adsorption

The toxin adsorption evaluation was carried out by using a total area of each nonwoven fabric of 60 cm^2^. The preparation of urea, inulin, and albumin solutions, the measurement, and the calculations were carried out as described in previous works [32].

## 3. Results

### 3.1. X-ray Diffraction (XRD)

**Figure 1:**
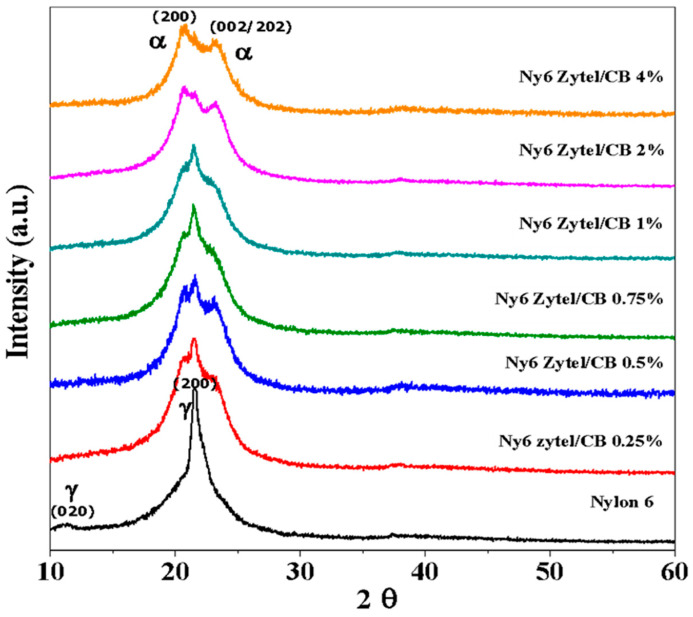
X-ray diffraction patterns of Nylon 6 nonwoven fabric and Ny6 Zytel/CB nonwoven fabric at different concentrations of MCBNs (0.25, 0.5, 0.75, 1.0, 2.0, and 4.0%).

Figure 1 shows the diffraction patterns of Nylon 6 and Ny6 Zytel/CB nonwoven fabrics at different loading percentages (0.25, 0.5, 0.75, 1.0, 2.0, and 4.0). Nylon 6 presents an intense signal at 21.58° and a weak signal at 11.23° of 2θ corresponding to the planes (2,0,0) and (0,2,0) of the crystalline form γ [42]. All nanocomposites exhibit significant changes in the crystalline structure of Nylon 6. The crystal structure diffractograms of Ny6 Zytel/CB composites show a mixture of α and γ crystalline phases: α with two intense diffraction peaks at 20.79 and 23.23° (planes 2,0,0 and 2,0,2/0,0,2 doublet, respectively) in 2θ scale and a single intense γ diffraction peak at 21.58° (plane 200) in 2θ scale (Figure 1) [43,44,45]. This change is related to the development of the α crystalline phase, which is intensified by increasing the concentration of nanoparticles in the nanocomposite. In the presence of MCBNs, the γ diffraction peak is less intense than the two α diffraction peaks that increase mainly at concentrations of 2 and 4% by weight. This transformation of the crystalline phase change from α to γ in Nylon 6 has also been observed in clinoptilolite (zeolite) composites [38]. Gururajan et al. [42] suggested that the crystalline phase change of Nylon 6 can be attributed to differences in the crystallization kinetics of the material. Likewise, Konishi et al. [46] suggested that the incorporation of carbon black in a nylon polymeric matrix improves diffraction peaks as the amount of carbon black inside the matrix increases, thus favoring the phases of the nylon structure. It is important to note that the CB must present broad diffraction peaks at 24.5° (intense) and 43.15° of 2θ; however, they are not appreciated, because they are found in a very small amount with respect to Nylon 6 [38]. On another hand, Lee et al. attributed the amorphous structure to the degree of activation of carbon black and the modification chemical [47]

### 3.2. Fourier Transform Infrared Spectroscopy (FTIR)

**Figure 2:**
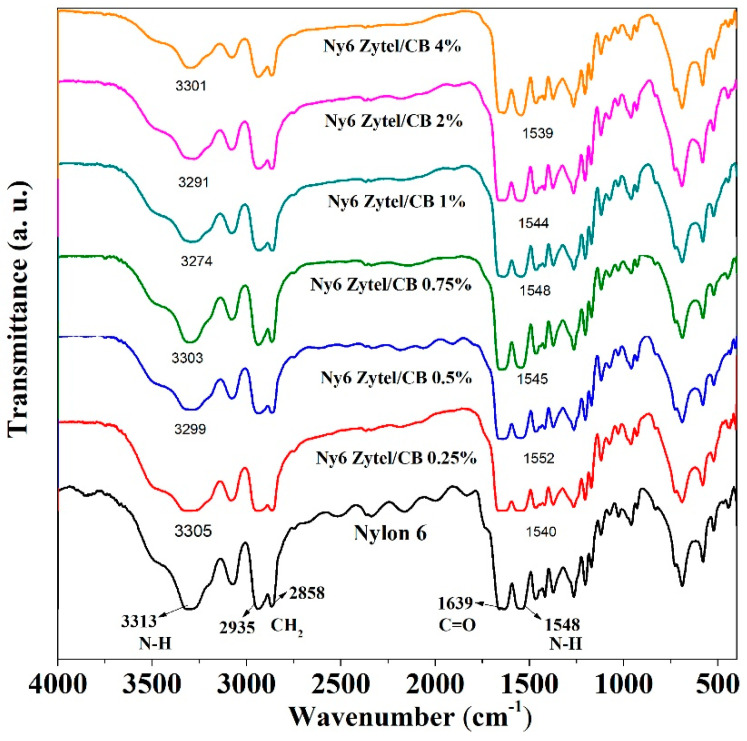
FT-IR spectra of Nylon 6 nonwoven fabrics and the samples obtained (0.25, 0.5, 0.75, 1.0, 2.0, and 4.0%).

FT-IR spectra of Nylon 6 nonwoven fabric and Ny6 Zytel/CB fabrics are shown in Figure 2. Absorption bands at 3313 and 1548 cm^−1^ are attributed to N-H bond stretching, and the last band is typical of secondary amine stretching due to the primary amine change to secondary amine [48,49]. The signal at 1639 cm^−1^ is assigned to the carbonyl group present in the Nylon 6 structure. Signals are observed at 2935 and 2858 cm^−1^ and attributed to symmetric and asymmetric vibrations of CH_2_ bonds corresponding to polyamide [50,51]. All Ny6 Zytel/CB nonwoven fabrics present a spectrum similar to pure Nylon 6; however, a small shift in the absorption bands of N-H and C=O can be seen, the behavior of which was also observed in another report [38].

### 3.3. Thermal Properties

**Figure 3:**
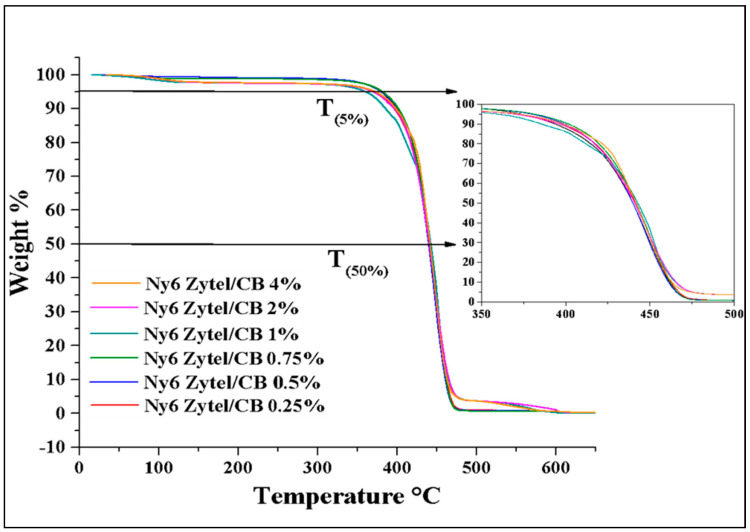
Thermogravimetric analysis of Ny6 Zytel/CB nonwoven fabrics at different concentrations (0.25, 0.5, 0.75 1.0, 2.0, and 4.0%).

Figure 3 presents the thermogravimetric analysis of Nylon 6 nonwoven fabric and each nonwoven fabric with modified CB. The results show that for each of the fabrics with MCBNs, a behavior similar to pure Nylon 6 is observed. In this process of decomposition, it can be observed that the first loss of mass starts in the range of 33 °C to 252 °C, which can be associated with the continuous loss of adsorbed water volatiles in the surface of the material and decomposition products of the organic part of MCBNs [52]. The second weight loss occurs in the range of 330 °C to 495 °C, which is attributed to the thermal degradation of Nylon 6 [38,43,51]. Subsequently, at temperatures of 330 to 495 °C, the second weight loss event is presented only for higher concentrations of particles (1, 2, and 4%), and this behavior is attributed to the reinforcement of modified carbon black present in Nylon 6.

A comparison analysis of all samples was performed between the temperature recorded with a weight loss of 5% (T_5%_) and a temperature of 50% (T_50%_) (Table 2). The results indicate that stability improves between 4 and 7 °C for nonwoven fabric with modified CB compared to pure nonwoven fabric Nylon 6. This behavior suggests that from concentrations above 1% of modified carbon black, the stability of the compound can improve. In previously reported work, they agreed that the incorporation of CB could provide Nylon 6 with greater thermal stability [39]. The experimental residue values at 550 °C are very close to those expected, and it is important to consider that carbon black has a 30% modification that degrades the MCBNs much earlier.

**Figure 4:**
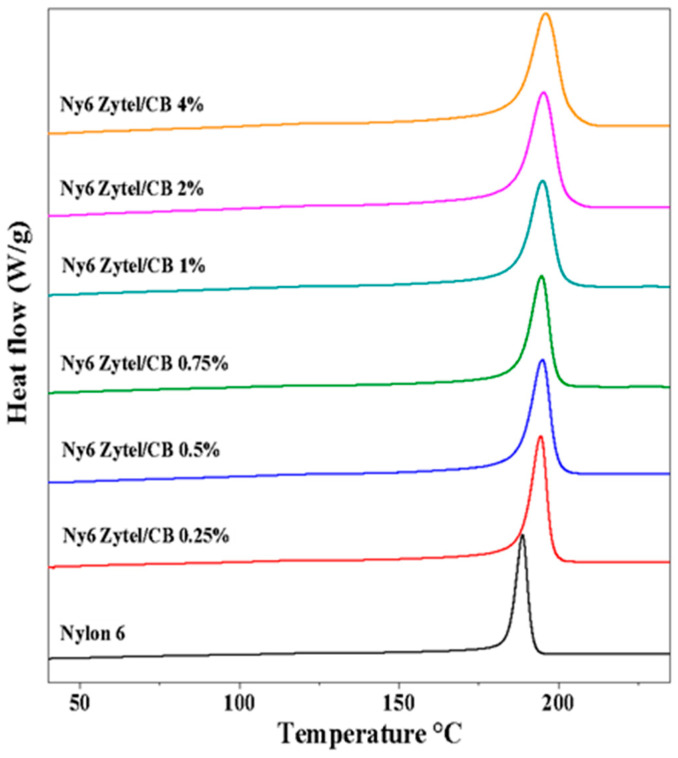
Crystallization exotherms obtained by DSC from Nylon 6 nonwoven fabrics and Ny6 Zytel/CB nonwoven fabrics at different concentrations (0.25, 0.5, 0.75, 1.0, 2.0, and 4.0 wt.%).

**Figure 5:**
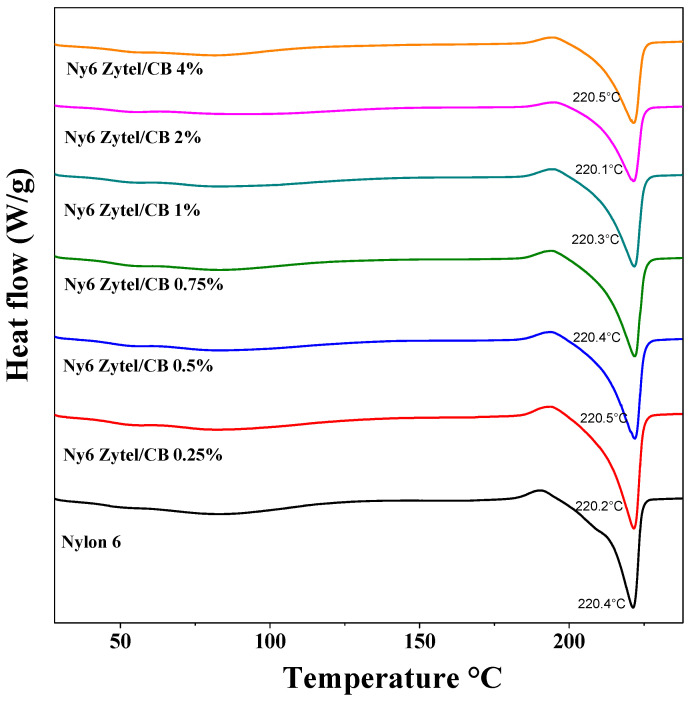
Melting endotherms obtained by DSC from Nylon 6 nonwoven fabrics and Ny6 Zytel/CB nonwoven fabrics at different concentrations (0.25, 0.5, 0.75, 1.0, 2.0, and 4.0 wt.%).

The crystallization and melting temperatures of the Nylon 6 nonwoven fabrics and samples obtained at different concentrations were evaluated by DSC (Figure 4 and Figure 5). The results of the crystallization process indicate that the crystallization temperature increases gradually with the charge increase of MCBNs, reaching temperatures of approximately 200 °C, which is higher than that of pure Nylon 6, suggesting an interaction between modified carbon black nanoparticles and the nylon amide groups; that is, there is a restriction on the mobility of nylon chains. The same behavior was observed in the fusion endotherms of nonwoven fabrics with modified CB [46,53].

The melting and crystallization enthalpies (Δ*H*) as well as the degree of crystallinity for each of the nanocomposites obtained are shown in Table 3. The degree of crystallinity (*X_c_*) was calculated using the following equation:

$$X_{c\;}\left(\%\right)=\frac{\Delta H_{f}}{\left(1-\emptyset \right)\Delta H^{*}}$$

where Δ*H_f_* is the heat of fusion of the sample; ΔH* is the heat of fusion of pure Nylon 6 with 100% crystallinity, equal to 230 J/g for the specific case of our polymer; Ø is the weight fraction of modified carbon black nanoparticles in the nanocomposite [45].

The results show that there are changes in the first melting temperature transition of pure Nylon 6 nonwoven fabric and nonwoven fabric of nanocomposites at different concentrations in a range of 0.77 to 2.74 degrees. These changes in the first melting transition depression are attributed to the nanometric size of MCB present in nylon fibers, as MCBNs have a much higher surface/volume ratio than nylon fibers, which alters their thermal properties. For the samples Ny6Zytel/CB4.0% and Ny6Zytel/CB2.0%, the first melting temperature transitions are 207.89 °C and 207.59 °C, respectively, obtaining the lowest values, while Ny6Zytel/CB0.75% reaches the highest value of all nanocomposites (T_f_ = 209.56 °C). Thermal properties such as melting enthalpy, crystallization temperature, and degree of crystallization of Ny6Zytel/CB nanocomposites are increased in comparison to pure Nylon 6 nonwoven fabric (see Table 3). The degree of crystallization for nanocomposites increases with increasing MCBN load, but for nanocomposites with high percentages of MCBNs (1–4 wt.%), this is not true; this behavior may be associated with the formation of particle aggregates that are not homogeneously dispersed in the continuous phase. Therefore, the nucleation process is less favorable and crystal growth is limited. In addition, the aggregates restrict the movement of the polymer chain, which makes it difficult to orient and pack the chains [54,55].

It is important to point out that the crystallinity remains high in those samples that have high MCBN contents (2 wt.% and 4 wt.%), which suggests that the new materials have good mechanical properties. According to previous studies, they report that these results could be attributed to the influence of the MCBNs in the Nylon 6 matrix, where the CB promotes the nucleation of the material [39,53].

### 3.4. Scanning Electron Microscope

**Figure 6:**
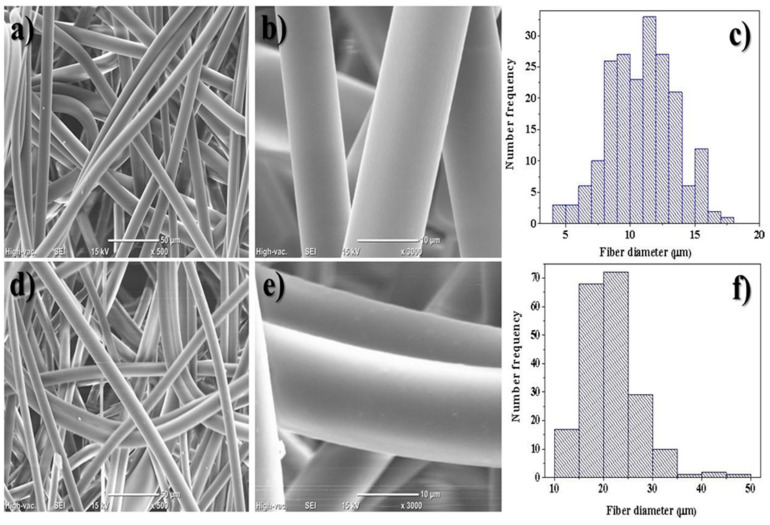
(**a**) Micrography of Ny6 Zytel/CB 0.25% nonwoven fabrics at 500×. (**b**) Micrography of Ny6 Zytel/CB 0.25% nonwoven fabrics at 3000×. (**c**) Histogram of size distribution of Ny6 Zytel/CB 0.25% fibers. (**d**) Micrography of Ny6 Zytel/CB 4% nonwoven fabrics at 500×. (**e**) Micrography of Ny6 Zytel/CB 4% nonwoven fabrics at 3000×. (**f**) Histogram of size distribution of Ny6 Zytel/CB 4%.

Figure 6 shows SEM micrographs and fiber size histograms of Ny6 Zytel/CB 0.25% and Ny6 Zytel/CB 4% nonwoven fabrics. The fibers show a uniform, smooth, and randomly oriented morphology. However, by increasing the load of modified carbon black particles, there is a greater interaction between the fibers compared to the lower concentration. The average fiber diameter distribution was evaluated from the measurement of approximately 200 fibers with different sizes of 5 to 50 µm. For Ny6 Zytel/CB material 0.25%, an average of 10.89 ± 2.56 µm was calculated, and for Ny6 Zytel/CB 4.0%, it was an average of 21.24 ± 5.51 µm. These results indicate that the increase in fiber diameter is attributed to the constant increase in material load (modified carbon black, MCB); similar studies have reported this behavior [3].

### 3.5. Contact Angle (Wettability)

**Figure 7:**
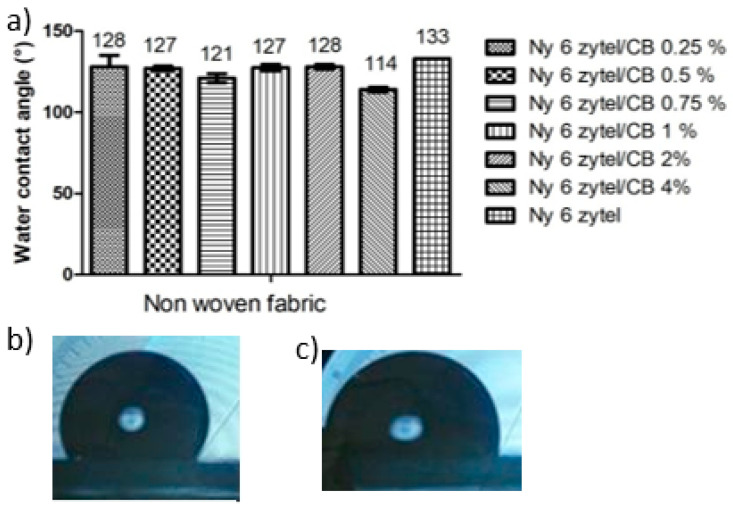
(**a**) Water contact angles of the samples Ny6 Zytel/CB to different con-centrations of carbon black and (**b**) images of water contact angles of the samples Ny6 Zytel pure (**b**) and Ny6 Zytel/CB 4% (**c**).

The measurement of the contact angle (θ_c_) allowed the characterization of the wettability of the surface of Nylon 6 nonwoven fabric and its respective fabrics with different concentrations of MCBNs. In the contact angle, the angle formed by a drop of water when it comes into contact with the sample is measured, in order to determine its hydrophobic or hydrophilic characteristics. The contact angles are dependent on the individual free surface energies of the solid and liquid, as well as the free energy of interaction between them. Figure 7a shows the results obtained of pure Nylon 6 and all nanocomposites, and Figure 7b shows the contact angle images of the pure Nylon 6 nonwoven fabric and the image of the Ny 6 Zytle/CB 4% nonwoven fabric. The pure Nylon 6 nonwoven fabric presents a contact angle θ_c_ = 133° ± 1, indicating that it has a hydrophobic characteristic, because there is a high surface tension energy because the cohesive forces of water are stronger than the adhesive forces [56]. All Ny6 Zytel/CB nonwoven fabrics have lower contact angles than pure Nylon 6, which suggests that the polar groups presence on the MCBN surface favors its interactions with the Nylon 6, so the new Ny6 Zytel/CB composites have a surface with more polar groups than pure Nylon 6, which leads to the formation of hydrogen bonds with H_2_O molecules. As mentioned above, MCBNs increase its adsorption capacity to water, and consequently, the nonwoven fibers of the nanocomposites are more wettable to water. It is also known that the superficial roughness increases when the functional groups increase [24]. The sample Ny6 Zytel/CB4.0% has the lowest contact angle value, θ_c_ = 114° ± 2, which suggests that increasing the concentration of nanoparticles increases the hydrophilic characteristic. This experimental evidence could increase the adsorption property of the material. That is, the surface tension energy decreases and adhesion forces increase, allowing the water drop to expand and, as a consequence, the contact angle to reduce. The values of contact angle of each sample are shown in the graph.

Ny 6 Zytel/CB 0.75% and Ny 6 Zytel/CB 4% samples exhibit the lowest contact angles (121° and 114°, respectively), suggesting that the Ny 6 Zytel/CB 4% is strongly affected by the amount of MCBNs present in the sample, making it the least hydrophobic. There are studies that suggest that the contact angle values also depend on an ensemble of partially impregnated and partially penetrated states in the top of the fabric, or over a limited fraction of the hemicylindrical upper part of the top textile fibers [57].

### 3.6. Adsorption of Uremic Toxins

**Figure 8:**
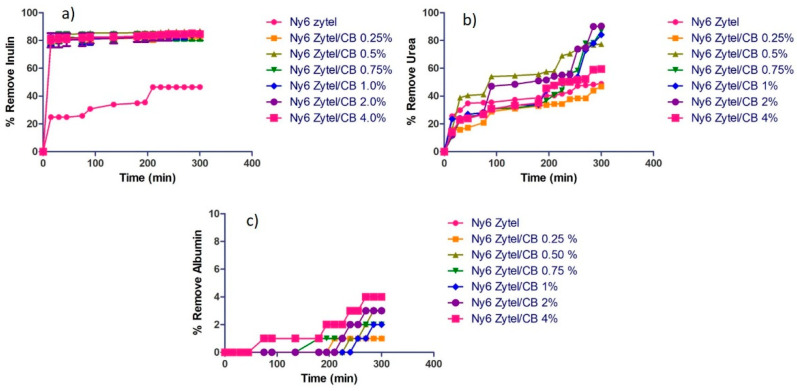
Removal percentage of (**a**) inulin, (**b**) urea, and (**c**) albumin.

Uremic toxins are those that result from renal retention in chronic kidney disease (CKD) and that contribute to the deterioration of multiple biochemical and physiological functions, which is defined as Uremic Syndrome. They also promote the progression of disease-damaged tubular cells; reports in the literature suggest that some toxins induce an oxidative stress [58]. Due to this, alternatives have been sought for the early detection of the initial phases in chronic kidney disease, as it occurs in many patients asymptomatically for a long time. Therefore, properly determining renal function is a priority; an exogenous substance that is indicative of renal glomerular function is inulin clearance, which is filtered by the kidney, not reabsorbed or secreted at the tubular level as other uremic toxins [59]. Figure 8 shows the percentage removal graphs of inulin, urea, and albumin using Nylon 6/MCBNs nonwoven fabric nanocomposites and pure Nylon 6 nonwoven fabric. Figure 8a shows that Nylon 6 nonwoven fabric has a removal rate of 46% inulin from 210 min. Nylon 6 nonwoven fabric nanocomposites with modified carbon black nanoparticles at different MCBN loads show high removal percentages in a range of 80–85% at 15 min of contact, which indicates great performance and efficiency in a very short time. These results could suggest that the time of hemodialysis treatment, which is 4 h, could be decreased according to the adsorption process of uremic toxins. Figure 8b shows the graph of the percentage of urea toxin removal, which can cause damage to tubular cells. Observed results indicate that Nylon 6 nonwoven fabric and Ny6 Zytel/CB 0.25% nonwoven fabric present a similar percentage of removal (48%), which could be due to the low amount of MCBNs present in the polymer matrix, while all other fabrics show a high percentage of urea removal (80 to 90%). The sample Ny6 Zytel/CB 2.0% has the highest urea removal percentage at 285 min. Similar studies have reported the same urea removal percentages using two types of carbon nanoparticles (carbon black and graphene nanoplatelets) [39]. The result in this research is relevant because only MCBNs were used and the use of higher-cost materials such as graphene was avoided. CRD causes in patients a protein-energy wasting, which is characterized by a loss of body protein mass as well as energy; a biomarker is albumin [60], which decreases in the process of hemodialysis. A low serum albumin concentration has also been well documented to be a predictor of mortality in hemodialysis patients [61,62]. This was the reason why the study of the adsorption process of nanocomposites was contemplated. Figure 8c shows that the loss of this protein is less than 6%; these findings could help in the treatment of hemodialysis, demonstrating the selectivity of nanocomposites obtained by the adsorption of certain toxins.

### 3.7. FT-IR after Adsorption

**Figure 9:**
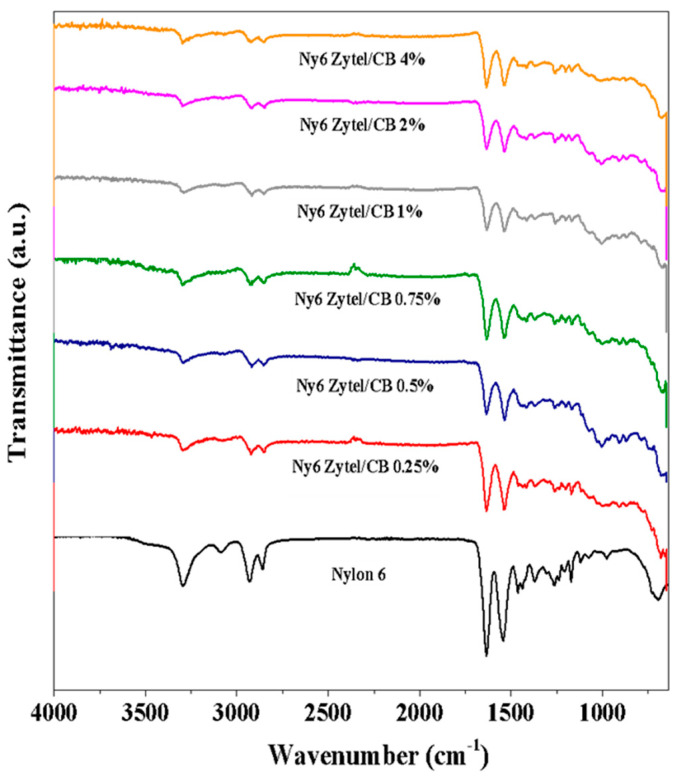
FT-IR spectra of Nylon 6 nonwoven and nonwoven fabrics at different concentrations (0.25, 0.5, 0.75, 1.0, 2.0, and 4.0%) after adsorption of uremic toxins.

Figure 9 shows all spectra of pure Nylon 6 nonwoven fabrics and Ny6 Zytel/CB fabrics at different concentrations after being evaluated as uremic toxin adsorption nanomaterials. In the spectra of nanocomposites, the adsorption bands characteristic of the chemical structure of Nylon 6 can be seen again, and some typical absorption bands of toxins (urea, albumin, and inulin) can be used in the evaluation of nanocomposites [63,64,65,66]. However, the signals of each of the uremic toxins interfere in their determination, because signal overlap occurs because the wavelengths are very similar between them and those of Nylon 6.

### 3.8. Analysis of the Findings Found in the Adsorption Performance of the Nanocomposite

For a better explanation of the results, it is essential to analyze the chemical structures of the adsorbent material and uremic toxin. Inulin is an intermediate-sized molecule that contains in its chemical structure several hydroxyl groups that are able to interact with the functional groups present of Nylon 6, achieving a removal of 46% of inulin in 210 min. The simple fact of introducing MCBNs considerably improves adsorption efficiency, which is explained by the greater hydrophilic characteristic that the nanocomposites present with respect to Nylon 6 (see Section 3.5). In addition, the monotonous behavior that inulin has is due to the fact that the adsorption sites are quickly occupied and there is no change over time. The process by which a porous solid is capable of retaining the particles found in an aqueous fluid can be carried out by a chemical adsorption involving the bonds of the modified carbon black or a physical adsorption, both of which can be carried out at the same time.

In the case of urea, the explanation is similar: there is a greater adsorption of the uremic toxin because the size is smaller compared to inulin and the steric effect must play an important role. Albumin adsorption is more limited; only 2.1% is adsorbed when using pure Nylon 6. It is the same when the Ny6 Zytel/CB nanocomposites are used: adsorption levels are still low. This can be explained by the high molecular weight of albumin (66,500 g/mol) and its negative charge that repels with the natural negative charge of carbon black. The zeta potential value of CB is −25 mV [67].

The MCBNs present in Nylon 6 alters the properties, and the ability of adsorption and selectivity in a process of adsorption of toxins is improved. This strategy of using resin-based adsorbents and nanoparticles has been used in recent years. In the case of carbon nanomaterials, the first thing sought is to modify the surface of the nanoparticles with polar groups and then process the nanocomposite (in the form of fiber, hollow fiber, nonwoven fabric, etc.) always. In order to have a high surface area, these new materials are also known as nanoporous adsorbents [38]. Nanoscale materials generally present high adsorption capacity. A good example where the effect of polar groups and particle size can be seen is described by Abidin et al., in developed and oxidized starch nanoparticles (oxy-SNPs) for urea adsorption, which presented a maximum adsorption capacity of 185.2 mg/g [68]. The best urea adsorption result of Ny6 Zytel/CB samples was 155.2 mg/g and is comparable with the highest reported values for the different urea adsorbents materials.

## 4. Conclusions

In this work, new Ny6Zytel/CB nonwoven fabrics were developed by melt-blowing and tested as adsorbent materials. Ny6Zytel/CB nonwoven fabrics showed a hydrophilic characteristic compared with the pure Nylon 6. The Ny6Zytel/CB nonwoven fabrics had optimal distribution, size, and very good uremic toxin removal in comparison to benchmark Nylon 6 nonwoven fabric. The inulin adsorption on the Ny6Zytel/CB nonwoven fabrics reached 80–85% in a short time (15 min), whereas the Nylon 6 nonwoven fabric had a nonlinear behavior in removal of the toxin, reaching 46% of removal in the same time. The urea toxin was removed to 80–90% using the Ny6Zytel/CB nonwoven fabrics. The use of Ny6Zytel/CB nonwoven fabrics for retaining the albumin was effective as the loss of this protein was less than 6%. The results indicate that for the treatment of hemodialysis, these Ny6Zytel/CB nonwoven fabrics could help it, as they demonstrated the selectivity of the adsorption of certain toxins.

## Figures and Tables

**Table 1:** Identification of nanocomposites.

Sample Identification	Specifications	Load Weight Percent (wt.%)
Nylon 6	Nonwoven fabric of Nylon 6 and modified carbon black 0 wt.%	0
Ny6 Zytel/CB 0.25%	Nonwoven fabric of Nylon 6 and modified carbon black 0.25 wt.%	0.25
Ny6 Zytel/CB 0.5%	Nonwoven fabric of Nylon 6 and modified carbon black 0.5 wt.%	0.5
Ny6 Zytel/CB 0.75%	Nylon 6 nonwoven fabric and modified carbon black 0.75 wt.%	0.75
Ny6 Zytel/CB 1.0%	Nonwoven fabric of Nylon 6 and modified carbon black 1.0 wt.%	1
Ny6 Zytel/CB 2.0%	Nonwoven fabric of Nylon 6 and modified carbon black 2.0 wt.%	2
Ny6 Zytel/CB 4.0%	Nonwoven fabric of Nylon 6 and modified carbon black 4.0 wt.%	4

**Table 2:** Thermal properties of Nylon 6 nonwoven fabrics and nonwoven fabrics with modified nanoparticles.

Sample	Temperature (°C) at Different Percentages of Weight Loss		Residue to 550 °C
	T_5%_ (°C)	T_50%_ (°C)	(%)
Nylon 6	372 ± 0.5	436 ± 1	0
Ny6 Zytel/CB 0.25%	370 ± 1	441 ± 0.5	0.39 ± 0.02
Ny6 Zytel/CB 0.5%	379 ± 0.7	440 ± 0.7	0.55 ± 0.07
Ny6 Zytel/CB 0.75%	380 ± 1.5	442 ± 0.2	0.69 ± 0.03
Ny6 Zytel/CB 1.0%	358 ± 1	443 ± 0.8	1.93 ± 0.19
Ny6 Zytel/CB 2.0%	367 ± 0.5	441 ± 0.8	2.22 ± 0.26
Ny6 Zytel/CB 4.0%	372 ± 0.6	442 ± 0.7	2.52 ± 0.04

**Table 3:** Melting enthalpy (J/g), crystallization enthalpy (Δ*H*, J/g), crystalline (*X_c_*) Nylon 6 nonwoven fabrics, and Ny6 Zytel/CB nonwoven fabrics to different concentrations of 0.25, 0.5, 0.75, 1.0, 2.0, and 4.0 wt.%.

Sample	First Transition (T_f_ °C)	Melting Enthalpy (J/g)	Crystallization Enthalpy(Δ*H*, J/g)	Degree of Crystallization (*X_c_*, %)
Nylon 6	206.82 ± 0.5	73.17	79.85	31.81 ± 2.18
Ny6 Zytel/CB 0.25%	209.56 ± 0.2	89.85	98.39	39.16 ± 2.58
Ny6 Zytel/CB 0.5%	209.22 ± 0.7	90.64	90.05	39.61 ± 2.58
Ny6 Zytel/CB 0.75%	209.43 ± 0.5	96.08	97.44	42.09 ± 2.64
Ny6 Zytel/CB 1.0%	208.73 ± 0.2	91.09	92.71	40.01 ± 2.43
Ny6 Zytel/CB 2.0%	207.59 ± 1	101.18	96.01	44.89 ± 2.69
Ny6 Zytel/CB 4.0%	207.89 ± 0.5	94.15	90.16	42.64 ± 1.6

± standard deviation from three measurements.
